# Lutein and cognition in children

**DOI:** 10.1017/jns.2014.54

**Published:** 2014-11-13

**Authors:** Billy R. Hammond

**Affiliations:** Brain and Behavioral SciencesThe University of GeorgiaAthens, GA 30602-3013USA

In a recent paper Mulder *et al.*^(^^1^^)^ analysed the relationship between measurements of cognition and dietary and plasma lutein levels in children. Mulder *et al.*'s correlational study was based on previous observations that lutein is found in the brain^(^^2^^)^ and has been linked to cognition and central nervous system function in adults^(^^3^^)^. Unlike previous work in adults, however, Mulder *et al.* found no relationship between their assessments of cognition and lutein measured in blood and diet. On this basis, they conclude that any effect of lutein on cognition in children is likely to be ‘subtle’ and difficult to separate from other covariates. Although this very well may be true, the data and results provided by Mulder *et al.* cannot be used as the basis for such a conclusion.

First, their study population was particularly well nourished, which would have reduced their ability to detect associations based on restricted range. For example, in a large US sample (US National Health and Nutrition Examination Survey; NHANES), the average intake of lutein for 4- to 8-year-olds was 311 (sd 474) μg/d^(^^4^^)^. Based on the same assessment (24 h recall), average intake of lutein in the Mulder *et al.* sample was nearly four times higher (mean intake was 2130 µg/d when assessed by their FFQ). As the authors note, this suggests ‘higher intakes of lutein among children in the present study…’. Like many food components, the largest functional effects of lutein may be the most significant for those with relative deficiency^(^^5^^)^. Since the authors did not supply the range of scores on the Kaufman Assessment Battery (KABC) or Peabody Picture Vocabulary Test (PPVT), it is impossible to evaluate whether similar statistical issues (restricted range) apply to their cognitive scores.

Another significant limitation of the study was the selection of plasma and diet as a biomarker for lutein in brain. Some of the most compelling data on the role of lutein and brain function come from studies that were not cited by the authors^(^^6^^–^^10^^)^. Those studies directly measured lutein in central nervous system tissue. The technology exists to measure lutein and zeaxanthin non-invasively within retinal tissue (there referred to as macular pigment; MPOD)^(^^11^^)^. This can even be done in premature infants^(^^12^^)^ and has been done in children^(^^13^^)^. Concentrations in the retina, being part of the central nervous system, are highly correlated with concentrations of lutein in the brain^(^^14^^)^. In contrast, diet and serum (as used by Mulder *et al.*) are known to not correlate well with central nervous system levels of lutein. For example, serum lutein tends to only explain about 7 % of the variance in retinal lutein; dietary intake only explains about 4 %^(^^15^^)^. It is unlikely that the plasma or dietary lutein assessments that Mulder *et al.* conducted predicted variation in central levels of this pigment in children. Indeed, as Mulder *et al.* note, when using the FFQ, dietary intake of lutein only explained about 10 % of the variation in serum lutein: repeated immediate recall of intake (three 24 h food diaries) only explained 25 % of the variance. Past studies have shown that serum lutein and zeaxanthin are only moderately predictive of serum lutein and zeaxanthin assessed at a separate time^(^^16^^)^. If serum lutein cannot strongly predict itself, it seems unlikely to be able to predict variation in the brain, especially in children.

The type of cognitive testing the authors performed (the KABC and PPVT) are very broad-ranging tests of developmental status. Such tests are probably not the most sensitive means of measuring the effects of diet on brain development. For example, recent studies on young college studies have shown significant effects of lutein when using more direct and atomistic assessments of neural activity such as visual processing speed^(^^17^^)^ or neuroimaging^(^^18^^)^.

We share the authors' view on the importance of nutrition on optimal brain development. It is unlikely, however, that the Mulder *et al.* study will help inform our understanding of whether lutein has an influence on brain function during this critical period. As the authors note, this population was at ‘low risk’ for ‘nutrient inadequacy’ and therefore enhancing cognition beyond average seems an unreasonable goal. Perhaps a better framing of the conclusion of this study would be that higher lutein intake in a nourished sample of children is unrelated to cognitive enhancement when measured using relatively coarse cognitive assessments.

There are no conflicts of interest to report.
